# Regulatory Science of Extracellular Vesicles: From ISEV Standards to Translational Implementation (Insights From Japan)

**DOI:** 10.1002/jev2.70358

**Published:** 2026-08-17

**Authors:** Yusuke Yoshioka, Yu Fujita, Takahiro Ochiya

**Affiliations:** ^1^ Department of Molecular and Cellular Medicine Institute of Medical Science Tokyo Medical University Tokyo Japan; ^2^ Division of Next‐generation Drug Development Research Research Center for Medical Sciences The Jikei University School of Medicine Tokyo Japan

**Keywords:** comparability, extracellular vesicle therapeutics, fit‐for‐purpose analytics, manufacturing control, regulatory science

## Abstract

Extracellular vesicles (EVs) offer compelling opportunities for diagnostics and therapeutics, yet translation toward routine clinical use and commercial readiness remains limited. In diagnostics, EV‐based assays may reach practice as laboratory‐developed tests, while in therapeutics unproven “exosome” offerings have prompted safety communications, together underscoring the need for fit‐for‐purpose regulatory science. Here, we organize implementation challenges into six recurring bottlenecks: Identity; Purity; Potency; Measurement comparability; Manufacturing control; and Safety. We summarize how international standardization efforts led by the International Society for Extracellular Vesicles (ISEV), including MISEV2023 and task‐force outputs, strengthen reproducibility and comparability, while highlighting remaining gaps that arise when descriptive guidance must be converted into operational specifications and submission‐ready evidence packages. Using Japan as a case example, we illustrate how regulatory‐authority discussions, professional‐society statements, and emerging implementation‐oriented guidance can clarify review questions, distinguish therapeutic EV products from conditioned medium, and support trust‐preserving translation. Finally, we propose a non‐binding, deliverables‐based roadmap across near‐, mid‐, and long‐term horizons to enable clinical and commercial readiness. The roadmap should be adapted to product context, risk profile, and jurisdiction, and it emphasizes precompetitive collaboration across academia, industry, and government to build shared infrastructure for measurement, standardization, reproducibility, and quality evaluation, thereby translating scientific consensus into operational development and review frameworks.

**Abbreviations**
AMED: Japan Agency for Medical Research and Development
CDMO: contract development and manufacturing organization
CMC: chemistry, manufacturing, and controls
CPP: critical process parameter
CQA: critical quality attribute
EU: European Union
EV: extracellular vesicle
FDA: U.S. Food and Drug Administration
GMP: Good Manufacturing Practice
IND: investigational new drug
ISEV: International Society for Extracellular Vesicles
IVD: in vitro diagnostic
JSEV: Japanese Society for Extracellular Vesicles
LDT: laboratory‐developed test
LoD: limit of detection
LoQ: limit of quantification
MISEV: Minimal Information for Studies of Extracellular Vesicles
NTA: nanoparticle tracking analysis
PEG: polyethylene glycol
PMDA: Pharmaceuticals and Medical Devices Agency (Japan)
QC: quality control
UK: United Kingdom

## 1 Why “Regulatory Science of EVs” and Why Now?

Extracellular vesicles (EVs) have attracted substantial attention as promising tools for both diagnostics and therapeutics (Kumar et al. 2024; Mizenko et al. 2024). Because EVs can be obtained from body fluids and carry diverse molecular cargo, they are being actively explored for applications such as liquid biopsy, as well as for therapeutic modalities including drug delivery and immunomodulation (Escudé Martinez de Castilla et al. 2021; Tamura et al. 2021). With rapid advances in basic research, knowledge of EV molecular features, functions, and in vivo behaviour has expanded, and an increasing number of studies are approaching clinical translation.

Despite this progress, however, relatively few EV‐related products have reached routine clinical use or commercial deployment, and a considerable gap remains between research‐stage technologies and “standard” tools in real‐world healthcare. In diagnostics, for example, tests based on exosome/EV‐derived RNA in urine (e.g., ExoDx) are often cited as being close to clinical implementation (Margolis et al. 2022); nonetheless, such tests are provided as laboratory‐developed tests (LDTs) and have not been established as cleared/approved in vitro diagnostics (IVDs). In this sense, EV‐based diagnostics can move toward clinical use, but still face challenges in fully demonstrating comparability and quality assurance within a formal regulatory framework. Although EV‐based diagnostics and therapeutics are often discussed together, the implementation barriers are not identical: diagnostics place particular weight on analytical validity, comparability, and quality assurance in routine testing environments, whereas therapeutics additionally require robust strategies for manufacturing control, potency, change management, and safety evaluation. For therapeutics, these requirements also make translational collaboration with contract development and manufacturing organizations (CDMOs) often important. In many cases, academic proof‐of‐concept workflows are not directly transferable to clinical development, and substantial redevelopment may be required to establish scalable downstream purification, fit‐for‐purpose analytics, and manufacturing control strategies that are compatible with real‐world product development. We emphasize that EV diagnostics remain an important translation domain, and that several fit‐for‐purpose and measurement‐comparability principles discussed here, such as sample handling, analytical performance characterization, and quality assurance concepts, are relevant across both diagnostics and therapeutics. However, because therapeutic translation typically requires a broader and more complex operational package, including CMC and GMP, potency strategies, manufacturing control and change management, and safety evaluation, this Perspective focuses primarily on EV therapeutics, while diagnostic‐specific regulatory pathways are discussed only at a high level.

In therapeutics, implementation pressures can also outpace regulatory and quality readiness. Unapproved “exosome products” have been marketed and offered as clinical interventions in some settings, prompting safety alerts from regulatory authorities. In Japan as well, administrative communications have been issued in response to the expansion of such offerings in self‐pay medical services, highlighting a gap between the momentum for implementation and the preparedness of regulatory and quality frameworks. These developments indicate that legitimate clinical development and unproven clinical interventions can coexist under the broad “exosome” label. From the standpoint of patient safety and maintaining trust in the field, establishing an appropriate regulatory‐science foundation is therefore an urgent priority.

A key contributor to this gap is that the regulatory science required to make EVs viable as “products” or “tests”, that is, a shared language that can withstand regulatory review and real‐world operation has not been sufficiently developed. In particular, descriptive characterization that may be acceptable in exploratory research does not automatically translate into operational definitions, acceptance criteria, release tools, or comparability frameworks required for development. Even if strong scientific evidence accumulates, translation will be hindered unless we can answer, in a fit‐for‐purpose manner, questions such as: What exactly is being defined as the target entity (Identity)? What impurities are acceptable, and to what extent (Purity)? How should biological activity be supported and controlled (Potency)? How can measurement traceability and cross‐laboratory/platform comparability be ensured (Measurement comparability)? How can manufacturing consistency and post‐change comparability be demonstrated (Manufacturing control)? And how should safety, including biodistribution, be evaluated within an appropriate study design (Safety)?

Accordingly, in Chapter 2 we translate these issues into “questions that are asked in regulatory review,” and organize them into a structured set of bottlenecks for EV translation. In Chapter 3 we summarize how international standardization efforts (including those led by ISEV) have provided shared foundations, and we clarify the remaining gaps that become critical at the implementation stage. In Chapter 4 we use Japan as a case example to extract practical lessons on translating global standards into an operational regulatory‐science framework, not because these challenges are unique to Japan, but because they illustrate, in a concrete and timely way, broader issues that are increasingly relevant across jurisdictions. In Chapter 5 we propose a practical roadmap, presented as a non‐binding set of options and deliverables, to accelerate responsible EV translation. Finally, in Chapter 6 we conclude with key takeaways and a call for coordinated efforts across academia, industry, and government.

This Perspective is timely because EV translation is entering a phase in which scientific maturation, implementation pressure, market expansion, and regulatory attention are progressing simultaneously, making previously tolerable ambiguities increasingly problematic for clinical development and public trust.

## 2 Bottlenecks for Productization and Implementation: Distinguishing “Research Barriers” From “Translation Barriers”

Challenges in translating EV‐based diagnostics and therapeutics are not simply a matter of “immature science.” In research, the priority is to ensure interpretability, that is, to be able to explain what is being analyzed and to avoid misattributing mechanisms or causality. In implementation, by contrast, the central question is whether clinically meaningful performance can be maintained in a reproducible, operationally feasible manner. This shifts attention to process robustness, throughput, cost, change management, and an overarching quality assurance framework that integrates manufacturing and safety. A major and often underappreciated barrier is that analytical methods widely used in exploratory EV research do not always provide the reproducibility, traceability, or control utility required for specification setting, lot comparison, and Good Manufacturing Practice (GMP)‐oriented manufacturing decisions (Lener et al. 2015). Accordingly, the “optimal” approach for research and the “optimal” approach for implementation may overlap, but they do not always coincide.

### 2.1 Workflow Design as Fit‐for‐Purpose

Selection of EV isolation and analytical workflows should be guided by the nature of the sample and the requirements of downstream use, including the desired balance between yield and specificity, that is, a fit‐for‐purpose approach. From an implementation perspective, the acceptable degree of co‐isolated materials, the necessary level of quality control (QC), and the allowable complexity of the workflow depend strongly on the context of use (e.g., diagnostics vs. therapeutics; discovery vs. routine operation; centralized laboratories vs. near‐patient settings). Importantly, fit‐for‐purpose must apply not only to the isolation workflow, but also to the measurement system itself: a method that is useful for exploratory particle detection or relative comparison may still be inadequate for defining specifications, dose metrics, or comparability thresholds needed for development (Lener et al. 2015). The key point is not to rank methods in the abstract, but to design workflows around performance, manageability, and operational constraints for a given use case.

### 2.2 Illustrative Examples of Divergence Between Research and Implementation: Isolation/Enrichment Choices and Particle Counting

As one illustrative example (among many) of how “research optimal” and “implementation optimal” can diverge, polyethylene glycol (PEG)‐based precipitation kits can enrich a broad range of components, which may be problematic in research settings where co‐isolated contaminants and uncertainty about what is being captured can compromise mechanistic interpretation. In contrast, for diagnostic implementation, requiring ultracentrifugation as a “gold standard” may itself be a major operational barrier. A simple enrichment step can reduce hands‐on time, improve throughput, and potentially enhance reproducibility in clinical laboratories. Therefore, feasibility for implementation should be judged not by preference for a method per se, but by clinical performance and analytical performance (e.g., reproducibility, robustness, interference, and inter‐laboratory concordance). Importantly, when co‐precipitated components can interfere with the downstream assay, implementation requires an explicit management strategy (QC items, acceptance criteria, and evaluation of lot‐to‐lot variability) aligned with the context of use. Beyond enrichment workflows, the measurement system itself can become a limiting factor for implementation.

A related but even broader implementation challenge concerns particle‐based measurement itself. Nanoparticle tracking analysis (NTA) is widely used in EV research as one practical approach to estimate particle counts and, in some settings, to inform dose setting, process monitoring, or recovery assessment. However, variability arising from instrument settings, operator handling, pre‐analytical conditions, and non‐EV background particles illustrates a broader issue: particle‐based measurements that are useful for laboratory‐level characterization may not yet be sufficiently robust, specific, or traceable to support specification setting or GMP‐oriented manufacturing control (Vestad et al. 2017; Bachurski et al. 2019). In this sense, the key issue is not NTA alone, but the analytical maturity of measurement systems used for high‐stakes development decisions.

### 2.3 Bottlenecks Framed as “Questions Asked in Regulatory Review” (Research vs. Implementation)

Below we organize recurring translation issues into six bottlenecks. For each, we contrast the main barrier in research with the corresponding “review‐and‐operation” question that arises in implementation. Across all six, a recurring cross‐cutting issue is analytical maturity: without measurement systems that are reproducible enough to support decision‐making, even well‐conceived biological or manufacturing strategies may fail to translate into implementable controls (Welsh, van der Pol, Bettin et al. 2020).

(1) **Identity**
*Research Barrier* EVs are heterogeneous, with continuous distributions across origin, size, surface molecules, and cargo. As a result, “what exactly is being analyzed” can become ambiguous. This ambiguity is often amplified because different isolation and measurement workflows emphasize different EV subpopulations, making the intended EV population implicitly method‐dependent.
*Implementation Barrier* For products and tests, identity must be operationalized rather than merely described. Developers need to define what constitutes the measurand or drug substance concept and specify objective criteria for “sameness” (QC and acceptance criteria) that can be maintained across lots and after process changes. In practice, developers may need an orthogonal set of identity attributes because no single marker can reliably capture product “sameness” across manufacturing changes and across analytical platforms. For EVs, this is particularly challenging because the analytical methods used to describe particle‐associated properties may not yet be sufficiently robust for specification‐level control (Welsh et al. 2024).
(2) **Purity**
*Research Barrier* Co‐isolated materials (e.g., protein complexes, lipoproteins, nucleic acids, polymers) and other co‐isolated impurities can distort interpretation and lead to incorrect attribution of function. This issue is often amplified because different isolation and pre‐analytical workflows bring different classes and amounts of co‐isolated material into the EV fraction, even when the starting sample is the same (Ter‐Ovanesyan et al. 2023).
*Implementation Barrier* Purity cannot be reduced to a single metric; instead, critical impurities should be identified and controlled within fit‐for‐purpose acceptance ranges. Because impurity classes and associated risks differ across applications, developers typically need orthogonal measurements and a context‐of‐use rationale to justify acceptance ranges rather than relying on a single purity score. In diagnostics, the primary concern is assay interference; in therapeutics, impurities are linked to safety risks such as immunogenicity, toxicity, and altered biodistribution.
(3) **Potency**
*Research Barrier* EV mechanisms of action are often multi‐factorial, making it difficult to establish functional assays that are reproducible and mechanistically interpretable (Nguyen et al. 2020). This limitation becomes evident when the same EV preparation is evaluated across different bioassay formats or recipient‐cell contexts, yielding divergent readouts that are difficult to reconcile mechanistically.
*Implementation Barrier* Potency must function as a release and consistency tool, not merely a “paper assay.” A potency strategy should connect surrogate readouts to process control so that lot‐to‐lot variability, stability over storage, and comparability after process changes can be evaluated in a practicable manner. In practice, developers must also justify why the selected potency assay is fit for purpose and how it links to product attributes and clinical intent, even when mechanisms remain incompletely defined and reference concepts are still evolving. This challenge is compounded when the denominator used to express EV input or dose, such as particle count, depends on measurement systems whose reproducibility, specificity, and traceability may still be insufficient for implementation‐grade control (Welsh, van der Pol, Bettin et al. 2020).
(4) **Measurement Comparability**
*Research Barrier* EV measurements are highly method‐dependent; differences in instruments, pre‐analytics, and analysis pipelines can yield different values from the same sample, undermining cross‐study synthesis. This variability is further compounded by the limited availability of widely adopted reference materials and harmonized calibration approaches, making it difficult to distinguish biological differences from measurement‐system effects.
*Implementation Barrier* Comparability is a foundation for maintaining clinical performance. Diagnostics require analytical performance characterization (precision and reproducibility, linearity, limit of detection (LoD) and limit of quantification (LoQ), interference, and inter‐laboratory agreement). Therapeutics likewise require a measurement system (calibration, references, and standardized records) that enables lots to be assessed along consistent axes. In regulated development, this comparability must be sustained across laboratories and over time, including after operational changes, which increases the need for traceable measurement records and fit‐for‐purpose reference concepts. In the EV field, however, methods widely used at the research level, such as particle counting by NTA in some settings, may not yet provide the level of reproducibility, traceability, specificity, or inter‐platform concordance needed for specification setting, dose definition, or GMP‐oriented control (Vestad et al. 2017; Bachurski et al. 2019). This gap between laboratory utility and implementation‐grade measurement remains a major bottleneck for translation, highlighting the need for stronger analytical frameworks, reference concepts, and fit‐for‐purpose control strategies.
(5) **Manufacturing Control**
*Research Barrier* Small differences in culture conditions, collection timing, and isolation steps can shift EV properties, harming reproducibility. Such shifts can arise from subtle changes in cell state and handling (e.g., confluency, medium composition, and processing timing) that are rarely matched identically across laboratories, leading to drift in EV composition and functional readouts (Palviainen et al. 2019; Bost et al. 2022; Borger et al. 2025).
*Implementation Barrier* Scale‐up and long‐term operation inevitably involve changes in materials, equipment, and process parameters. Because EV products can be sensitive to upstream and downstream conditions, even well‐intended process optimization may alter critical attributes, increasing the need for pre‐specified comparability plans and decision rules. A structured approach to comparability is therefore essential (Lener et al. 2015). Manufacturing consistency must be supported by linking critical quality attributes (CQAs) to process controls and by establishing change management procedures to handle deviations and process evolution. In practice, achieving this often requires close interaction with CDMOs or other manufacturing partners, because the transition from an academically optimized workflow to a development‐ready process may require redesign of purification steps, analytical packages, and scalability assumptions rather than simple transfer of an existing protocol (Thakur and Rai 2024). A further translational challenge concerns the producer‐cell strategy. In some natural EV programs, early research may rely on primary cells, whereas longer‐term clinical and commercial development may require more sustainable producer‐cell platforms. This raises difficult questions about how cell‐source changes, expansion strategies, or immortalization approaches affect product identity, safety, and regulatory acceptability, particularly when comparability must be demonstrated across changes in the producer‐cell platform.
(6) **Safety**
*Research Barrier* Results can vary substantially across animal species, routes of administration, and dose definitions; furthermore, limitations of labelling and detection methods complicate interpretation of biodistribution (Kang et al. 2021). In addition, incomplete labelling controls and the risk of free‐label or non‐EV background signals can make it difficult to attribute observed biodistribution patterns to intact EVs.
*Implementation Barrier* Safety evaluation must be tailored to product risk (e.g., immunogenicity, coagulation‐related effects, off‐target accumulation) and supported by an explainable biodistribution assessment. In practice, developers often need a pre‐specified, risk‐based safety package that links biodistribution findings to dose metrics, route of administration, and impurity profiles so that safety conclusions remain interpretable across lots and after process changes. The impact of manufacturing‐derived impurities and lot‐to‐lot variability on safety should also be addressed in an integrated manner. Safety assessment may also need to take into account the biological and manufacturing history of the producer cells, particularly when immortalized or otherwise engineered cell substrates are used to support scalability (Xia et al. 2024). In such settings, developers may need to justify how producer‐cell characteristics are controlled and why the resulting EV product remains acceptable from both quality and safety perspectives.
  Table 1 maps these six bottlenecks to implementation‐grade deliverables and indicates where each topic is addressed throughout this Perspective. In the next chapter, we summarize how international frameworks (including those led by ISEV) have addressed these bottlenecks to date, and we clarify the remaining gaps that become critical for implementation.

**TABLE 1:** Translating shared guidance into implementation‐grade deliverables for EV translation.

Bottleneck	Research barrier	Implementation barrier	Selected guidance/examples	Implementation‐grade deliverables	Where addressed in this Perspective
Identity	EV heterogeneity and workflow dependence can make the intended EV population and measurand ambiguous.	Identity must be operationalized with orthogonal attributes and “sameness” criteria that remain stable across lots and after process changes.	MISEV2023; Biofluid TF papers (blood/urine/CSF); Single‐EV FCM reporting guidance	Working measurand or drug‐substance concept; identity specification and “sameness” criteria (QC and acceptance concepts)	Ch2.3(1); Ch5(S2); Ch4.2–4.4
Purity	Co‐isolates and contaminants vary by workflow and can distort functional attribution.	Purity requires risk‐based impurity identification and acceptance ranges supported by orthogonal measurements.	MISEV2023; Biofluid TF papers (blood/urine/CSF)	Critical‐impurity concept; acceptance ranges and rationale (interference and safety relevance)	Ch2.3(2); Ch5(S3); Ch5(M1)
Potency	Multifactorial mechanisms complicate reproducible and interpretable functional assays.	Potency must support release and comparability by linking surrogate readouts to process control and dose metrics.	ISEV clinical trials position paper; EV functional assay/potency frameworks	Potency strategy as a release and comparability tool; dose‐metric alignment and decision‐grade readouts	Ch2.3(4); Ch3.4; Ch5(S5); Ch5(M3)
Measurement comparability	Method dependence and limited reference concepts make cross‐study synthesis difficult.	Decision‐grade measurement needs traceable records, inter‐lab concordance, and comparability after operational changes.	MISEV2023; Single‐EV FCM reporting guidance; Biofluid TF papers; NTA variability/accuracy studies	Reference and calibration concepts; measurement qualification plan; inter‐lab agreement and comparability strategy	Ch2.3(4); Ch3.4; Ch5(S5); Ch5(M3)
Manufacturing control	Small upstream and downstream differences can shift EV properties and functional readouts.	Scale‐up requires CQA–CPP linkage and comparability‐based change management, often involving CDMOs.	ISEV clinical trials position paper; ICH Q5E; ICH Q8–Q10	CQA–CPP linkage; change control; post‐change comparability package and decision rules	Ch2.3(5); Ch5(M1); Ch5(M3); Ch5(L2); Ch4.2–4.4
Safety	Species, route, and dose differences plus labeling and detection limits complicate biodistribution interpretation.	Risk‐based safety packages must link biodistribution, dose metrics, and impurity profiles to maintain interpretability across lots and after changes.	ISEV Safety Notice; FDA safety communications; BD evidence syntheses	Implementable safety and biodistribution plan; interpretability controls; cell‐substrate history considerations	Ch2.3(6); Ch5(M4); Ch5(L3); Ch4.1–4.4

Abbreviations: BD, biodistribution; CDMO, contract development and manufacturing organization; CMC, chemistry, manufacturing, and controls; CPP, critical process parameter; CQA, critical quality attribute; EV, extracellular vesicle(s); FCM, flow cytometry; GMP, Good Manufacturing Practice; ICH, International Council for Harmonisation of Technical Requirements for Pharmaceuticals for Human Use; NTA, nanoparticle tracking analysis; TF, task force.

## 3 What International Frameworks Have Advanced and What Remains Unresolved: ISEV's Standardization Portfolio and the Bridge to Regulatory Science

Successful clinical and commercial translation of EV‐based diagnostics and therapeutics requires more than accumulating promising biological findings. It also requires a shared foundation that improves reproducibility, comparability, and interpretability across studies and products, so that quality, safety, and efficacy can be discussed in a common language. Over the past decade, ISEV has played a central role in building this foundation through community‐driven guidance, including successive updates of the Minimal Information for Studies of Extracellular Vesicles (MISEV) guidelines (most recently MISEV2023), as well as position papers, and task‐force activities (Lötvall 2024; Welsh et al. 2024). At the same time, a shared language for describing EVs does not automatically provide analytically mature measurement tools for specification setting, dose definition, or manufacturing comparability.

### 3.1 Beyond MISEV2023: Moving From General Recommendations to Context‐Specific Guidance

MISEV2023 provides a broad, field‐wide baseline for EV nomenclature, pre‐analytical variables, separation and characterization, and in vitro/in vivo studies (Welsh et al. 2024). However, the bottlenecks that matter most for translation often emerge in highly context‐dependent ways, depending on biofluid type, analytical technology, and the intended context of use. In response, ISEV and its task forces have supported a growing portfolio of more specialized community outputs, including position papers and “minimal information” style documents tailored to specific sample types (e.g., blood (Lucien et al. 2023), urine (Erdbrügger et al. 2021), cerebrospinal fluid (Sandau et al. 2024)) and specific measurement modalities (e.g., single‐EV flow cytometry (Welsh, van der Pol, Arkesteijn et al. 2020; van der Pol et al. 2022)). The practical value of these documents is that they do not simply list “recommended experiments,” but instead map major sources of variability across the end‐to‐end workflow across pre‐analytics, isolation/enrichment, measurement, and reporting, so that reproducibility and cross‐study comparability can be improved systematically rather than piecemeal.

### 3.2 From Standardization to Regulatory Science: The Scope of ISEV's Regulatory Affairs Task Force

Standardization is a necessary foundation for translation, but it does not automatically resolve the questions that arise when EVs are treated as investigational products. Reflecting this, ISEV's Regulatory Affairs Task Force (focused on the regulatory affairs and clinical use of EV‐based therapeutics) explicitly targets the interface between EV science and real‐world development, including identifying relevant regulatory guidance and its application to EVs as investigational new drugs (INDs), and supporting safe and effective EV‐based treatment concepts globally (ISEV 2021). In practice, this “translation layer” is where topics such as potency strategies, manufacturing scale and GMP considerations, and reporting expectations for engineered/modified EVs become central, not as research preferences, but as development necessities that must connect manufacturing control to clinical intent. This bridge is especially important in areas where commonly used research measurements may not yet have the analytical maturity required for regulatory decision‐making, lot comparison, or comparability assessment (Welsh, van der Pol, Bettin et al. 2020).

### 3.3 The Real‐World Pressure Test: Unproven “Exosome” Interventions and the Need for Safety Communication

The need for regulatory science is not driven only by scientific uncertainty; it is also driven by the reality that “exosome” has become a marketing label that can outpace evidence and regulation (Asadpour et al. 2023). ISEV's Patient Information and Safety Notice on extracellular vesicles/exosomes and unproven therapies directly addresses this issue by communicating uncertainties and risks to patients and the public (ISEV 2020). Regulatory authorities have issued similarly direct warnings. For example, the U.S. Food and Drug Administration (FDA) Public Safety Notification on Exosome Products states that there are currently no FDA‐approved exosome products and emphasizes that claims that such products fall outside drug/biologic regulation are incorrect (FDA 2019). Media reporting also illustrates how quickly “exosome” labelling can spread beyond conventional medical channels; for instance, reports in the United Kingdom (UK) have described cosmetic clinics offering human‐cell‐derived “exosome” treatments despite prohibition under relevant UK/ European Union (EU) regulatory constraints, raising concerns about product authenticity and potential infectious risks (Devlin 2025). These developments underscore an uncomfortable but important point: in EV therapeutics, the translation pathway is shaped not only by what is scientifically possible, but also by how the field manages risk communication, public trust, and clear distinctions between evidence‐based development and unproven interventions.

### 3.4 The Remaining Gaps: From “Shared Language” to Implementable Evidence Packages

Taken together, ISEV's portfolio has substantially strengthened the shared foundations for EV research by improving reporting, reducing variability, and clarifying best practices across the workflow. Yet several gaps remain when the same knowledge must be translated into implementable development and review packages. In particular, moving from descriptive guidance to operational specifications (acceptance criteria), establishing potency strategies that function as practical release and comparability tools, building measurement traceability and inter‐laboratory agreement suitable for routine use, and designing change management/comparability frameworks for scale‐up and process evolution are challenges that are not fully resolved by standardization alone. A particularly important unresolved issue is analytical maturity of measurement systems: methods that are highly useful for exploratory EV characterization or relative comparison may still be insufficiently robust for dose definition, lot release, specification setting, or post‐change comparability in real‐world development. In addition, the broader ecosystem, including patient safety communication and the differentiation of legitimate clinical development from unproven “exosome” marketing has become an essential part of responsible translation. Scientific standardization, analytical maturity, and trust‐preserving communication can be viewed as complementary pillars rather than separate agendas (FDA 2019; ISEV 2020). Regulatory authorities have also taken enforcement actions against firms marketing “exosome” products with disease‐related claims outside approved pathways, including FDA warning letters (FDA 2025).

In the next chapter, we use Japan as a case example to extract practical lessons on how global standards and regulatory‐science concepts can be translated into an operational framework under real‐world constraints, and we connect these lessons to a proposed, non‐binding roadmap for responsible EV translation.

## 4 Insights From Japan: Translating Regulatory Science for EV Therapeutics Into an Operational Framework

International standardization has provided a shared foundation for EV research, but real‐world translation of EV therapeutics requires an additional operational layer to enable clinical and commercial readiness, integrating regulatory pathways, quality systems (chemistry, manufacturing, and controls (CMC)/GMP), safety expectations, and clinical operational constraints. In this chapter, we use Japan as a case example to extract practical lessons on translating global standards into an operational regulatory‐science framework, and we connect these lessons to the roadmap proposed in the next chapter. We focus on Japan's Pharmaceuticals and Medical Devices Agency (PMDA) framing, the Japanese Society for Extracellular Vesicles (JSEV) statement, and their English‐language synthesis, while detailed coverage of other initiatives is beyond the scope of this manuscript.

Throughout this chapter, we use “EVs (extracellular vesicles)” as an umbrella term. We retain “exosome” only when quoting formal wording in regulatory documents or widely used public‐facing labels, noting that the term may be used with varying definitional rigor. We also distinguish conditioned medium (which contains EVs and many other components) from therapeutic EV products.

### 4.1 “Implementation Momentum” Can Outpace Preparedness of Quality and Regulatory Frameworks

A notable feature of the current EV therapeutics landscape is that clinical interest and market momentum can grow faster than the establishment of robust evidence and quality systems. In Japan, administrative communications have been issued in response to cases where products derived from human cell culture materials, sometimes described as “reagents”, were marketed in ways that could imply therapeutic use, despite the absence of formal confirmation of quality, efficacy, and safety as regulated medicinal products. Importantly, these communications emphasize that, at present, there are no approved medicinal products marketed as “exosome‐related” products under the relevant regulatory framework. Such developments highlight a practical challenge for EV therapeutics: legitimate clinical development and unproven clinical interventions may coexist under the same broad label, creating risks for patient safety and undermining public trust in the field. This reality makes regulatory science both a technical discipline and a trust‐preserving infrastructure, an essential component of responsible translation. Importantly, these concerns should not be reduced to a simple binary of “regulated” versus “unregulated”. In practice, EV‐related activities may fall across different legal and operational domains, and implementation challenges often arise from how distinctions are made among regulated medicinal products, clinical practice, and commercial marketing and promotion.

### 4.2 PMDA Discussions Make the “Review Questions” Explicit

Japan provides an instructive example of how regulatory‐science questions for EV therapeutics can be made explicit and visible. Within the PMDA Science Board framework, a subcommittee dedicated to therapeutic products based on EVs (including “exosomes”) has been established (PMDA 2026). The subcommittee discusses evaluation of safety against viruses and other infectious factors, quality characterization of active ingredients, ensuring consistency in manufacturing, manufacturing process control, evaluation strategies for non‐clinical studies, and points to consider in clinical trials. In the PMDA Science Board report (provisional translation), these discussion themes are further organized into the following topics: (i) safety evaluation for infectious agents (e.g., viruses), (ii) evaluation of quality characteristics for active components, (iii) assurance of manufacturing consistency, (iv) manufacturing process control, (v) strategic considerations for nonclinical evaluation, and (vi) key considerations for clinical studies (PMDA 2023).

This scope aligns closely with the “questions asked in regulatory review” outlined in Chapter 2. Safety for infectious agents is not an isolated endpoint; it is tightly coupled to manufacturing process control and impurity management. Likewise, quality characteristics of the active component directly connect to identity, purity/impurities, and potency strategies. They also connect, in practical development, to questions about producer‐cell selection and control, particularly when a program must move from primary‐cell research workflows toward more sustainable manufacturing platforms capable of supporting clinical and commercial supply. The value of this PMDA‐led framing is that it shifts the discussion from “recommended experiments” toward a development‐and‐review logic, in which quality and safety must be articulated in a manner that can support consistent manufacturing, comparability after changes, and clinically meaningful interpretation. From an implementation standpoint, the value of this framing lies not in assuming that all analytical or methodological challenges have already been solved, but in clarifying which quality and safety questions must be addressed in development and how they should be linked to manufacturing and clinical intent.

### 4.3 The JSEV Statement Clarifies Boundaries and Social Prerequisites

In parallel, the Japan Society for Extracellular Vesicles (JSEV) has issued a statement that is highly relevant to regulatory science, not merely as a cautionary message but as an effort to clarify conceptual and practical boundaries (JSEV 2023). The statement highlights that efficacy evaluation for EV‐based clinical practices has not been completed at present and that therapeutic EV products used in clinical settings should be subject to rigorous review under the relevant regulatory framework. It further notes that clinical services sometimes advertised under labels such as “exosome therapy” may lack sufficient scientific validation.

A particularly important point for implementation is the clear distinction drawn between therapeutic EV products and conditioned medium (or other crude preparations) that may contain EVs alongside many other bioactive components (e.g., cytokines). This distinction matters for multiple bottlenecks simultaneously:

● **Identity**: what is the actual substance being administered or regulated as the therapeutic entity?
● **Purity**: how are co‐existing components, including impurities and co‐isolates, handled and controlled, and what is the rationale for acceptance ranges?
● **Manufacturing control**: what level of process control and quality system is necessary if the product is intended as a regulated therapeutic?
● **Trust‐preserving communication**: unapproved or poorly characterized interventions can damage confidence in the entire domain, slowing legitimate development.

From a regulatory‐science perspective, this type of boundary‐setting functions as a “social prerequisite” for translation: it supports clearer communication about what is evidence‐based development versus what remains unproven, and it encourages alignment between scientific claims, manufacturing reality, and clinical intent. It also helps clarify that the evidentiary and quality expectations for a regulated therapeutic product are fundamentally different from those for less well‐defined or insufficiently characterized preparations.

### 4.4 An English‐Language Synthesis and Movement Toward Practical Implementation Guidance

A distinctive feature of the Japanese case is that regulatory‐science discussions have been synthesized and made accessible internationally. In association with the PMDA subcommittee's outputs, Takakura et al. provide an English‐language review that organizes key quality and safety considerations for therapeutic products based on EVs along the development workflow: production, purification, characterization, quality evaluation and control, safety assessment, and clinical development/evaluation (Takakura et al. 2024).

From the perspective of this manuscript, this review can be positioned as an effort that builds on the foundational shared language established by community guidance, while pushing one step further into the operational issues required for product development and regulatory review (e.g., CMC/GMP considerations, process control, and the logic linking nonclinical/clinical design to quality and safety). This “translation layer” is precisely what becomes critical when EV therapeutics are treated as investigational products rather than research materials. At this stage, the practical value of such implementation‐oriented work lies not in offering a single definitive analytical solution, but in providing a structured framework for how available measurements, their limitations, and their intended uses can be integrated into development and review. The same is true for process and cell‐substrate decisions: implementation‐oriented guidance is valuable not because it eliminates all uncertainty, but because it helps developers organize how purification redesign, manufacturing transfer, and producer‐cell strategy should be justified as part of a coherent development program.

In addition, Japan is moving toward more implementation‐oriented guidance through a Japan Agency for Medical Research and Development (AMED)‐supported, multi‐stakeholder (academia–industry–government) working group, which has compiled a draft guidance document focused on quality assurance for native (non‐intentionally modified) EV medicinal products derived from human cell sources (often referred to as “exosomes” in public‐facing contexts). Publicly available materials have indicated that this draft would proceed through public consultation and subsequent governmental publication processes; accordingly, as of 17 March 2026, the draft guidance has been released for public comment via the Japanese government's e‐Gov public comment system (comment period through 16 April 2026) (e‐Gov 2026a; e‐Gov 2026b). Once released, such a document may serve as a practical reference for developers by translating core regulatory‐science questions into implementable expectations (e.g., characterization strategies, impurity control concepts, specification setting, stability, and comparability considerations).

### 4.5 Lessons Learned: What Can be Generalized Beyond Japan

The Japanese experience should be interpreted cautiously. It does not demonstrate that the translational challenges of EV therapeutics have already been solved, nor has it yet resulted in an approved EV therapeutic product. Rather, its current value lies in showing how a scientific community and regulatory‐science framework can begin to make the relevant questions visible: what should be defined as the product, what attributes should be controlled, how quality and safety concerns should be framed, and how unverified “exosome” interventions should be distinguished from evidence‐based therapeutic development. In this sense, the Japanese case provides an example of agenda‐setting and boundary‐setting, rather than a completed regulatory model.

The Japanese case suggests several lessons that are likely generalizable across jurisdictions:

(1) **Early clarity on classification and pathway reduces downstream rework**.
  When a modality is new, uncertainty about “what it is” in regulatory terms can propagate into uncertainty about what evidence is required. Making the review questions explicit early helps align CMC, nonclinical, and clinical strategies.

(2) **Risk communication is part of responsible translation**.
  In fields where marketing labels can outpace evidence, maintaining a clear distinction between legitimate development and unproven interventions is essential to preserve trust and enable ethical clinical progress. In Japan, the JSEV statement contributed to this boundary‐setting by clarifying that EV‐based medical interventions, including those described as “exosome” therapies, should not be presented as established evidence‐based treatments unless supported by appropriate scientific, quality, and safety evaluation.

(3) **“Research optimal” and “implementation optimal” should be designed separately, then bridged**.
  Mechanistic clarity and minimal contaminants are critical for research interpretation, whereas operational feasibility and reproducibility under quality systems become dominant in implementation. Fit‐for‐purpose design must address both.

(4) **Operationalization matters: specifications and change management are the bridge**.
  Shared language is necessary but not sufficient. Translation requires converting descriptive knowledge into operational controls: specification setting, comparability after changes, and consistent decision rules that link manufacturing to clinical intent.

(5) **Staged operationalization is more realistic than waiting for perfect methods**.
  In emerging modalities such as EV therapeutics, translation cannot depend on the prior existence of fully mature methods for every attribute. A practical approach is to define what can be measured reliably at each stage, make limitations transparent, and progressively strengthen control frameworks as development advances.
  In this view, the goal of regulatory science is not to wait for a single perfect assay or standard, but to enable responsible development while methods, comparability frameworks, and expectations continue to evolve.

(6) **Producer‐cell strategy is a translational issue, not merely a research choice**.
  For native EV therapeutics, producer‐cell selection has consequences beyond biological activity. As development progresses from exploratory studies to clinical and commercial planning, scalability, supply continuity, manufacturing control, and safety considerations may require reconsideration of the original cell source. Producer‐cell transitions, including expansion strategies or immortalization approaches, therefore become a central comparability and regulatory‐science challenge rather than a purely technical matter.

These lessons also illustrate both the strengths and the current limitations of Japan's approach. The PMDA Science Board report helped organize quality, safety, manufacturing, nonclinical, and clinical considerations into a structured set of review questions for EV‐based therapeutics, and the English‐language review by Takakura et al. made this domestic discussion accessible to the international EV community. However, these efforts should be viewed as an initial step rather than as evidence that implementation‐level challenges have been resolved. Guidance documents, position statements, and regulatory‐science reports can clarify the questions that need to be asked, but they do not by themselves define product‐specific specifications, validate potency assays, establish dose metrics, or determine how comparability should be demonstrated after manufacturing changes. These elements must be justified within each development program and refined through regulatory consultation, review experience, and accumulation of shared case examples. This limitation should not be interpreted as a shortcoming of the Japanese approach, but rather as a reflection of the current maturity of the EV therapeutic field, in which many critical attributes remain product‐ and context‐dependent. Taken together, these lessons illustrate how global standards can be translated into an operational regulatory‐science framework under real‐world constraints. While institutional details differ across jurisdictions, the underlying challenge is not unique to Japan: many regions have faced the need to distinguish evidence‐based development from loosely defined “exosome” offerings and to connect scientific standards to product‐oriented expectations for quality, safety, and clinical accountability. For example, safety communications on unapproved exosome products in the United States illustrate that boundary‐setting between unsupported clinical claims and regulated product development is a shared issue. In Europe, broader frameworks for advanced therapies similarly emphasize the need to translate biological plausibility into product identity, quality control, nonclinical and clinical evidence, and comparability considerations. Thus, the Japanese case may be most useful internationally not as a jurisdiction‐specific template, but as a case study of how early regulatory‐science dialogue can clarify the questions that any EV therapeutic product is likely to face across regulatory systems. In the next chapter, we integrate these points into a practical, non‐binding roadmap, organized as deliverables across near‐, mid‐, and long‐term horizons, to support responsible EV therapeutics translation.

## 5 A Practical Roadmap Toward Clinical and Commercial Readiness: Proposed Deliverables for EV Therapeutics (Non‐Binding)

### 5.1 Positioning and How to Use this Roadmap

The roadmap presented in this chapter is not intended to replace or represent official requirements or positions of regulatory authorities (e.g., PMDA), professional societies (e.g., JSEV), or any specific organizations. Rather, it reflects the authors’ perspective on plausible options and discussion points that may support responsible EV therapeutics translation. In practice, development and review expectations can vary depending on the product concept, indication, risk profile, manufacturing approach, measurement systems, and jurisdiction; therefore, product‐specific strategy development, including engagement with relevant authorities, is essential.

**FIGURE 1:**
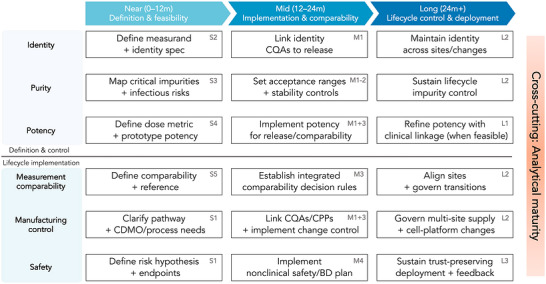
Integrated roadmap matrix linking six bottlenecks to staged deliverables for EV therapeutics. The figure summarizes how six recurring bottlenecks, Identity, Purity, Potency, Measurement comparability, Manufacturing control, and Safety, can be operationalized through near‐term (0–12 months), mid‐term (12–24 months), and long‐term (24+ months) deliverables toward clinical and commercial readiness. Each row corresponds to one bottleneck, and each cell indicates a practical objective for that stage. S, M, and L codes correspond to the Chapter 5 deliverables. The cross‐cutting theme of analytical maturity highlights that implementation‐grade measurement systems are required across all bottlenecks to support specification setting, dose definition, lot release, and comparability‐based change management.

The intent of this roadmap is to convert the six bottlenecks articulated in Chapter 2 (Identity; Purity; Potency; Measurement comparability; Manufacturing control; and Safety) into deliverables that can form an implementable evidence package. A fit‐for‐purpose approach remains central: priorities and depth of evidence should be adapted to the context of use and risk. As summarized in Figure 1, we propose a staged, deliverables‐based roadmap across near‐, mid‐, and long‐term horizons, structured as an integrated matrix that links the six recurring bottlenecks to staged implementation deliverables. Figure 1 should therefore be read as a matrix rather than as a simple chronological checklist. Each row corresponds to one bottleneck, and the near‐, mid‐, and long‐term columns indicate how the corresponding objectives may mature from definition and feasibility, to implementation and comparability, and finally to lifecycle control and deployment. In this framework, Table 1 summarizes the relationship among bottlenecks, barriers, community guidance, and implementation‐grade deliverables, whereas Figure 1 visualizes how those deliverables can be organized over time. The proposed timelines are indicative and non‐binding, and should not be interpreted as fixed regulatory milestones or universally applicable schedules. Importantly, the purpose of this roadmap is not to imply that all key methods or standards are already mature, but to provide a practical structure for development under conditions where measurement capabilities, comparability frameworks, and broader expectations for implementation are still evolving (Welsh et al. 2024).

### 5.2 Near Term (e.g., 0–12 Months): Define, Measure, and Quantify Variability

**Goal**: establish the minimum “definition + measurement + variability baseline” to reduce downstream rework.

● **(S1) Provisional pathway and development concept**. Summarize the product concept (source, degree of modification, route of administration, risk hypothesis) and clarify key questions for early regulatory interactions, including whether future development may require CDMO partnership and whether the process and producer‐cell strategy will need to be re‐engineered to support scalability and supply.
● **(S2) Provisional identity specification**. Define the working measurand or drug‐substance concept and propose orthogonal attributes that can support “sameness” criteria (QC and acceptance concepts), while flagging which of these attributes are sufficiently reliable for use in subsequent development stages.
● **(S3) Impurity and infectious‐risk mapping**. Identify candidate critical impurities and infectious‐agent risks, and map where removal and detection are expected within the process.
● **(S4) Prototype potency strategy**. Avoid reliance on a single assay; propose candidate surrogate readouts linked to process control, together with an initial dose metric definition.
● **(S5) Minimal comparability design**. Define core measurement records and repeatability and reproducibility checks, plus a calibration and reference concept to enable future inter‐lab comparisons, and identify early which assays are suitable for continued use and which may require further qualification or eventual replacement.

### 5.3 Mid Term (e.g., 12–24 Months): Demonstrate Consistency and Comparability Under Change

**Goal**: build a development system that can explain lot consistency and comparability through scale and process evolution.

- **(M1) CQA‐CPP linkage and release testing concept**. Identify candidate CQAs, connect them to critical process parameters (CPPs) and process controls, and propose acceptance and release testing concepts.
- **(M2) Stability, storage, and transport evidence**. Characterize how identity and potency change with time and handling, and connect those behaviours to specifications.
- **(M3) Change management and comparability framework**. Pre‐specify how process changes (scale‐up, materials, equipment) will be evaluated, including integrated decision rules across multiple metrics, while recognizing that comparability conclusions must be interpreted in light of the strengths and limitations of the underlying measurement system.
- **(M4) Implementable nonclinical safety and biodistribution plan**. Tailor endpoints to risk (e.g., immunogenicity, coagulation‐related effects, off‐target accumulation) and account for limitations of labelling and detection methods.
- **(M5) “Submission‐ready” documentation set**. Organize manufacturing records, analytical methods, specifications, deviation handling, and validation plans into a review‐ready package.

### 5.4 Long Term (e.g., 24+ Months): Sustain Readiness at Clinical and Commercial Scale

**Goal**: maintain accountability for quality and clinical intent at scale, across sites, and over time.

● **(L1) Mature potency strategy**. Improve the potency approach so it functions as a practicable release and comparability tool, and when feasible, relate it to clinical outcomes.
● **(L2) Quality system for multi‐site supply and long‐term operation**. Strengthen supply‐chain controls including source materials, inter‐site alignment of measurement and manufacturing, and long‐term change management, including sustained control of producer‐cell attributes and clear governance for any future manufacturing‐site changes or producer‐cell platform transitions.
● **(L3) Trust‐preserving implementation**. Reinforce risk communication, distinguish evidence‐based development from unproven interventions, and establish mechanisms for post‐deployment safety feedback.

## 6 Summary

This roadmap provides a non‐binding way to structure discussions and evidence development for EV therapeutics, translating recurring bottlenecks into practical deliverables across near‐, mid‐, and long‐term horizons. The roadmap should be adapted to the product's context of use and risk profile, while leveraging shared foundations from international standardization efforts and lessons from jurisdiction‐specific implementation experiences.

## 7 Conclusion: Toward Responsible EV Therapeutics Translation

EVs hold substantial promise for both diagnostics and therapeutics. Yet moving from scientific feasibility to clinical and commercial readiness requires more than compelling biological findings. It requires the ability to explain quality, safety, and efficacy in a shared language that can withstand regulatory review and real‐world operation. In this Perspective, we organized key translation barriers into six recurring bottlenecks (Identity; Purity; Potency; Measurement comparability; Manufacturing control; and Safety) and highlighted that “research optimal” and “implementation optimal” may not always coincide, underscoring the importance of fit‐for‐purpose design.

We also discussed how international standardization efforts led by ISEV have strengthened shared foundations for EV research through MISEV2023 and a broader portfolio of task‐force activities, position papers, and guidance documents (Welsh et al. 2024). At the same time, we emphasized that standardization alone does not fully resolve implementation‐critical challenges, such as operational specification setting, practical potency strategies that support release and comparability, analytically mature measurement systems that can support inter‐laboratory agreement and decision‐making, and change management frameworks for scale‐up and process evolution. In addition, the ecosystem surrounding EV therapeutics, particularly risk communication and clear differentiation between evidence‐based development and unproven “exosome” interventions, has become an essential part of responsible translation (FDA 2019; ISEV 2020).

Japan provides an instructive case example of how regulatory‐science questions can be made explicit and operationalized. These questions extend beyond analytical characterization alone and include how academic processes are converted into scalable manufacturing workflows, how CDMO‐linked development is organized, and how producer‐cell strategies are justified as products move toward clinical and commercial use. PMDA‐led discussions have clarified key considerations spanning quality characteristics, safety, including infectious‐agent risk, manufacturing consistency and process control, and strategic approaches to nonclinical and clinical evaluation. In parallel, the JSEV statement has contributed to boundary‐setting and social prerequisites for translation by emphasizing rigorous review for therapeutic EV products and by distinguishing therapeutic EV products from conditioned medium or other crude preparations that may contain many bioactive components beyond EVs. English‐language syntheses and evolving multi‐stakeholder efforts toward practical implementation guidance further illustrate how foundational concepts can be translated into development‐relevant considerations.

Importantly, advancing EV translation in practice will require coordinated efforts across academia, industry, and government. Foundational needs emphasized by international guidance, including MISEV, such as measurement and standardization, reproducibility, and quality evaluation, are difficult to establish and sustain by academia or pharmaceutical companies alone. A precompetitive approach, in which clinical requirements, industrial quality systems, and regulatory expectations are iteratively aligned, can help build shared infrastructure and evidence‐generation practices that benefit the field as a whole and support broader progress in EV drug development. This includes not only shared concepts and reporting expectations, but also practical approaches for using, qualifying, and improving measurements as part of real‐world development. In Japan, frameworks to promote such multi‐stakeholder, precompetitive capacity building have recently begun to take shape, including examples supported by public funding, and may serve as a useful case for enabling international standards to function effectively in real‐world implementation.

Finally, the roadmap presented here is non‐binding and reflects the authors’ perspective on plausible deliverables that can support clinical and commercial readiness. It should be adapted to the specific product concept, context of use, and risk profile, and refined through engagement with relevant authorities and stakeholders. By strengthening shared foundations, operationalizing key evidence packages, and reinforcing trust‐preserving practices, the field can move EV therapeutics from promising concepts toward responsibly developed medical technologies, while building the manufacturing, cell‐substrate, and comparability strategies needed for sustainable clinical and commercial translation.

## Author Contributions

Yusuke Yoshioka: conceptualization, writing – original draft, writing – review and editing. Yu Fujita: writing – review and editing. Takahiro Ochiya: writing – review and editing, supervision.

## Funding

No specific funding was received for the preparation of this manuscript.

## Conflicts of Interest

The authors declare no financial conflicts of interest. All three authors participate as members of an AMED‐supported working group related to EV guidance development, and one author serves as a member of a PMDA Science Board subcommittee related to EV‐based therapeutic products; these roles do not involve personal financial benefit, and the views expressed in this manuscript are those of the authors.

## Data Availability

Data sharing not applicable to this article as no datasets were generated or analysed during the current study.
